# T-Tau and P-Tau in Brain and Blood from Natural and Experimental Prion Diseases

**DOI:** 10.1371/journal.pone.0143103

**Published:** 2015-12-02

**Authors:** Richard Rubenstein, Binggong Chang, Robert Petersen, Allen Chiu, Peter Davies

**Affiliations:** 1 Department of Neurology, SUNY Downstate Medical Center, Brooklyn, New York, United States of America; 2 Department of Pathology, Case Western Reserve University, Cleveland, Ohio, United States of America; 3 Litwin-Zucker Center for Research in Alzheimer's Disease, Feinstein Institute for Medical Research, Manhasset, New York, United States of America; Rocky Mountain Laboratories, NIAID, NIH, UNITED STATES

## Abstract

Synaptic abnormalities are prominent in prion disease pathogenesis and are responsible for functional deficits. The microtubule associated protein, Tau, binds to and stabilizes microtubules in axons ensuring axonal transport of synaptic components. Tau phosphorylation reduces its affinity for microtubules leading to their instability and resulting in disrupted axonal transport and synaptic dysfunction. We report on the levels of total Tau (T-Tau) and phosphorylated Tau (P-Tau), measured by highly sensitive laser-based immunoassays, in the central nervous system and biofluids from experimentally transmitted prion disease in mice and natural cases of sporadic Creutzfeldt-Jakob Disease (sCJD) in humans. We found that, in contrast to sCJD where only the levels of T-Tau in brain are increased, both T-Tau and P-Tau are increased in the brains of symptomatic mice experimentally infected with the ME7, 139A and 22L mouse-adapted scrapie strains. The increased levels of T-Tau in sCJD brain, compared to control samples, were also observed in patient plasma. In contrast, there was no detectable increase in T-Tau and P-Tau in plasma from symptomatic experimentally infected mice. Furthermore, our data suggests that in mice showing clinical signs of prion disease the levels and/or ratios of T-Tau and P-Tau are only a useful parameter for differentiating the mouse-adapted scrapie strains that differ in the extent of disease. We conclude that the neuropathogenesis associated with P-Tau and synaptic dysfunction is similar for at least two of the mouse-adapted scrapie strains tested but may differ between sporadic and experimentally transmitted prion diseases.

## Introduction

Prion diseases, or transmissible spongiform encephalopathies (TSEs), are fatal, transmissible neurodegenerative disease in animals and humans. Prion diseases in animals include scrapie (sheep and goats), chronic wasting disease (deer and elk) and bovine spongiform encephalopathy (BSE) in cattle. The human diseases include Creutzfeldt-Jakob disease (CJD) [of which there are several types: sporadic (sCJD), familial, iatrogenic (iCJD) and variant (vCJD)] and Gerstmann-Straussler Scheinker syndrome (GSS), a familial prion disease whose neuropathology bridges prion disease and Alzheimer's disease (AD). Scrapie is the prototypical TSE and has been studied extensively in laboratory models of mice and hamsters. It is well established that TSE agents exhibit strain variation [1]. This has been observed for TSEs in several species, but has been most thoroughly documented for TSEs experimentally isolated in mice. The methods used for TSE strain discrimination have traditionally been based on simple observations of disease characteristics in strains of mice having the host gene (initially labelled *Sinc* but later identified as *PRNP*, the gene coding for the prion protein, PrP) responsible for short incubation periods (s7 or Prnp^a^) vs. long incubation periods (p7 or Prnp^b^). Many distinct strains of scrapie have been identified in mice of the same genotype and between different genotypes (Prnp^a^ vs. Prnp^b^), differing in their incubation periods, neuropathology, clinical features, weight changes, altered glucose tolerance, biochemical and physicochemical properties [2–10].

The most useful characteristic is the length of the incubation period from initial infection to the development of clinical disease, followed by the type and distribution of neuropathological changes seen in the brains of infected animals [2, 11, 12]. TSE strains have also been found to differ in their clinical manifestations, their ease of transmission to new species and their susceptibility to inactivation by heat and chemicals. The biochemical properties of disease-associated forms (PrP^Sc^) of the host PrP (PrP^C^) have also provided further criteria for distinguishing between TSE strains. Formal strain typing protocols in mice, based on incubation periods and neuropathology, have been used extensively as research tools.

The link between synaptic pathology and neurological deficits has been studied in ME7-infected mice where a progressive decrease in the number of synapses in the stratum radiatum was associated with degeneration of the presynaptic compartment and loss of dendritic spines, well before the death of CA1 pyramidal neurons [13–16]. Concomitant with this initial synaptic pathology, there are abnormalities in hippocampal synaptic plasticity, which parallel alterations in spontaneous etiological behaviors such as open field activity, burrowing, and nesting [17].

Further, studies of the ME7 scrapie strain in infected mice [18] demonstrated that slight changes in Tau metabolism and hyperphosphorylation are associated with scrapie during the late stages of disease (20 weeks) suggesting only that Tau dysfunction is not associated with the processes linked to early onset of disease and is therefore not a useful biomarker for early detection of TSEs.

The microtubule associated protein Tau is mainly expressed in CNS neurons and plays an important role in stabilizing microtubules, axonal maintenance and axonal transport. Although the accumulation of hyperphosphorylated Tau in the cerebral cortex is not a general feature of human prion disease neuropathology, it has been found in human cases and experimental mouse models of vCJD as well as in patients with a specific *PRNP* point mutation causing GSS [19, 20].

In this manuscript we compared the levels of total Tau (T-Tau) and phosphorylated Tau (P-Tau) in brain and blood from three experimentally transmitted mouse scrapie strains. These three scrapie agent-mouse strain combinations have well-characterized and distinguishing biological, biochemical and neuropathological properties. In addition, we also studied Tau in naturally occurring prion disease by measuring T-Tau and P-Tau in brain and blood from human cases of sporadic CJD (sCJD). Although quantitatively and qualitatively the levels of Tau only distinguished one of the three different mouse-adapted scrapie strains from the other two in clinical C57Bl/6J mice, the question still exists as to whether Tau-associated neuropathogenesis differs between naturally occurring forms of human prion diseases and those arising by transmission.

## Results

Weanling C57Bl/6J mice were infected by the intracerebral (i.c.) route with 1% brain homogenates (approximately 5 logs of infectivity) prepared from mice who were clinically affected with mouse-adapted scrapie (15 mice i.c. injected). The incubation periods of the infected C57Bl/6J mice were: 162 ± 8 for ME7, 137 ± 5 for 139A and 151 ± 5 for 22L. The neuropathology was similar between strains (data not shown), but the 139A strain demonstrated white matter involvement while ME7 and 22L did not [21]. In addition, all three scrapie strains contained minimal, if any, plaque formation.

The prion disease status of the infected mice was confirmed by Western blotting, which demonstrated the presence of PK-resistant PrP^Sc^ (PrP^res^) (not shown). The PrP^res^ profile was typical for PK-treated PrP^Sc^ showing three bands in the 20–30 kDa region comprised of unglycosylated (20–21 kDa), monoglycosylated (23–25 kDa) and diglycosylated (27–30 kDa) PrP. In addition, similar to our previous report [22], we observed that the PK resistance differed for PrP^res^ from the three scrapie strains (data not shown) with PrP^res^ isolated from ME7 and 22L brains having similar modest levels of PK sensitivity while PrP^res^ from 139A brains was partially digested to the three characteristic bands in the shortest time and thus was the most sensitive to PK digestion.

We hypothesized that the distinguishing biological and biochemical properties associated with neurodegeneration related to each group of scrapie strain-infected C57Bl/6J mice might be reflected in the levels of T-Tau and P-Tau in both the brain and blood at the late stages of clinical disease.

**Fig 1:**
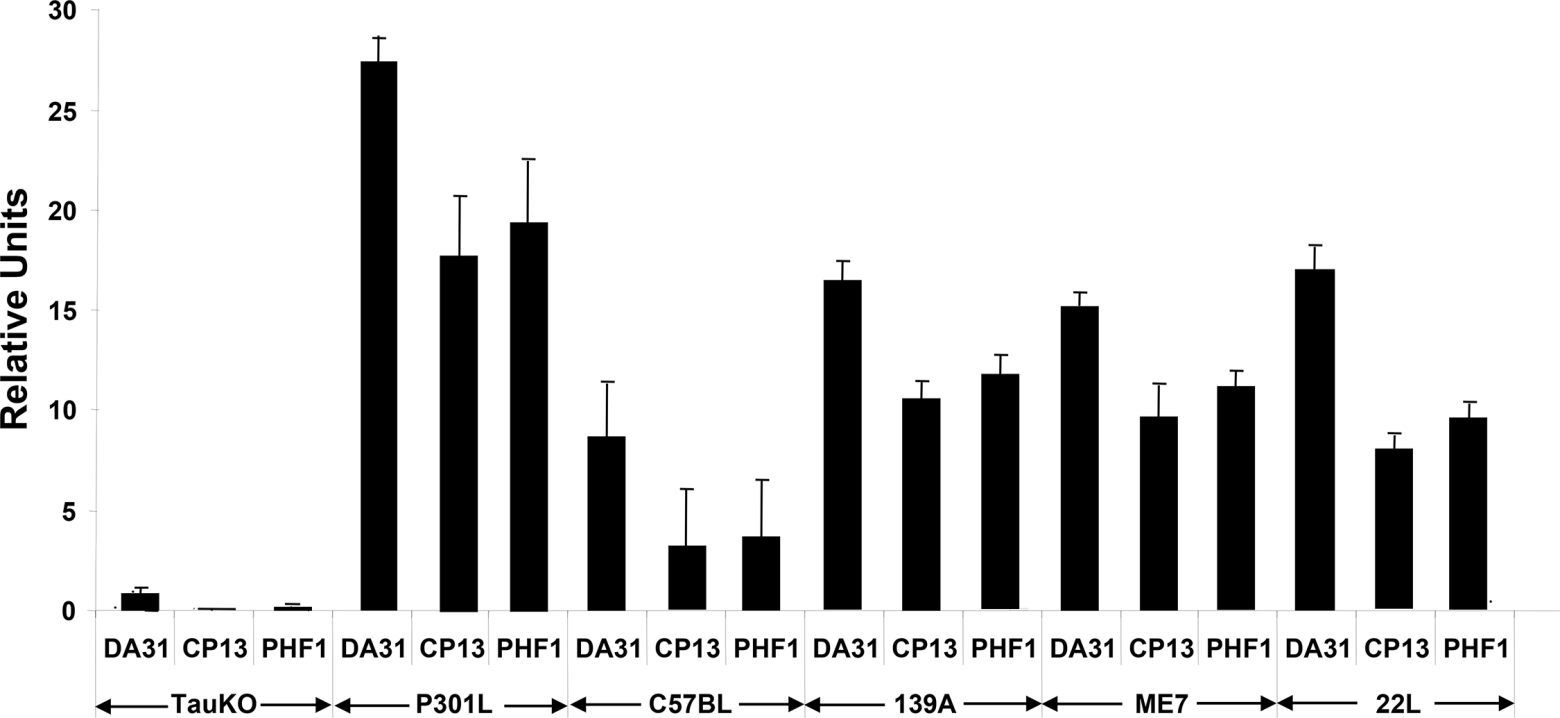
Quantitation of T-Tau (Mab DA31) and P-Tau (Mabs CP13 and PHF1) by EIMAF in uninfected, ME7, 139A and 22L scrapie strain-infected C57Bl/6J and P301L mouse brains. Uninfected TauKO mouse brains are used to demonstrate assay-related background Tau levels. All analysis was performed using a 10^−8^ dilution of brain lysates. Data is plotted as mean ± SD. Significance of T-Tau and P-Tau values between uninfected C57Bl vs. ME7-infected C57Bl mice is determined by the unpaired t-test (p<0.05). No significance differences, as determined by the unpaired t-test, was found in the T-Tau levels between ME7, 139A and 22L infected mice, but the P-Tau values of ME7 and 139A > 22L-infected mice (p<0.05).

We measured the levels of T-Tau (Mab DA31) and P-Tau (Mabs CP13 and PHF1) in the brains of uninfected C57Bl mice and three mouse-adapted scrapie strain-infected mice using the EIMAF (Enhanced Immunoassay using Multi-Arrayed Fiberoptics) technology [23] to achieve an accurate and quantitative assessment. Brains from uninfected TauKO mice [24] were used to establish the background levels of T-Tau and P-Tau signaling in our system, while the Tau overexpressing JNPL3 mouse line, a well characterized transgenic model of tauopathy which has a P301L mutation in the Tau gene, and develops neurofibrillary tangles, was used as a positive control [25]. Analysis of brain samples showed that scrapie-infected mice exhibited increased T-Tau and P-Tau levels compared to uninfected mice (Fig 1). The P-Tau/T-Tau ratio was significantly greater in the ME7 infected mouse brains compared to the uninfected C57Bl brains (~0.7 vs 0.3) (p < 0.05). When the brains from scrapie-infected C57Bl mice were compared, T-Tau was highest in the 139A and 22L infected mouse brains although not significantly different than in the ME7 infected brains. The P-Tau levels were similar in the 139A- and ME7-infected mice, but were significantly (p<0.05) lower in the 22L infected samples (Fig 1). This indicated that the biological differences between scrapie strains only altered the P-Tau levels.

**Fig 2:**
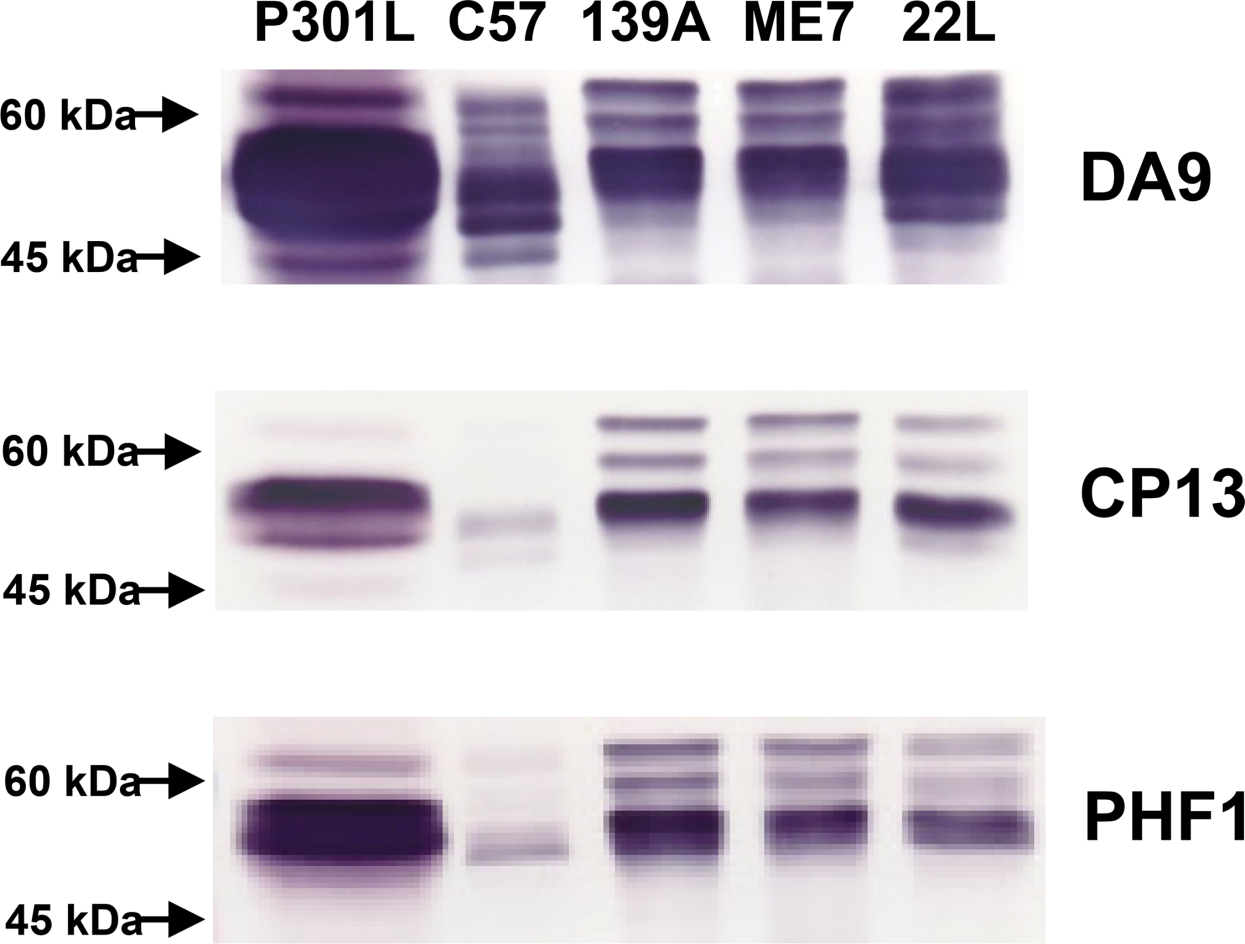
Western blot analysis of T-Tau and P-Tau from the brains of control (P301L and C57Bl) and scrapie agent-infected mice. Immunoblotting was carried out for T-Tau (Mab DA9) and P-Tau (Mabs CP13 and PHF1).

We examined the T-Tau and P-Tau profiles in uninfected and infected mouse brains by Western blotting. T-Tau was comprised of several major and minor proteins migrating in the 50–60 kDa region (Fig 2, Mab DA9). Western blot analysis of brain samples indicated that the T-Tau isoform profile of the 22L infected mice was similar to uninfected mice, whereas the 139A and ME7 mice showed more evidence of hyperphosphorylated forms (Fig 2, DA9), which may reflect the reduced volume of affected tissue in the 22L mice. The level of P-Tau was determined by immunostaining with Mab CP13 or Mab PHF1 (Fig 2) followed by densitometric analysis. As expected for the control mice, the level of P-Tau was highest in the P301L brains and least in the C57Bl controls. Of the scrapie-infected mouse brains, the amount of P-Tau was greatest in 139A followed by ME7 and then 22L, although the differences were not significant (p>0.05) when Mab CP13 was used for immunostaining, but were significant p<0.05) when Mab PHF1 was used to compare the three scrapie strains (Fig 2).

**Fig 3:**
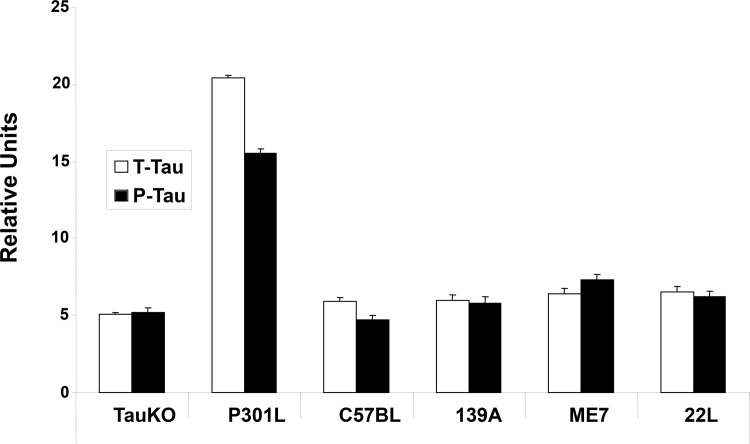
Analysis of T-Tau and P-Tau by a-EIMAF in plasma from ME7, 139A and 22L scrapie strain-infected mice. Tau levels in plasma from P301L and C57Bl/6J are shown as controls. Data is plotted as mean ± SD. No significance differences, as determined by the unpaired t-test, was found between T-Tau and P-Tau values of C57Bl uninfected vs. ME7, 139A or 22L-infected C57Bl mice.

Plasma levels of T-Tau and P-Tau were measured using a-EIMAF, defined as EIMAF in combination with rolling circle amplification, in the uninfected and scrapie-infected mice, uninfected TauKO mice and uninfected P301L mice (Fig 3). As expected, the plasma levels of T-Tau and P-Tau in the uninfected P301L mice were highest. However, in contrast to the findings in brain, the plasma levels of T-Tau and P-Tau in all the infected clinical mice, regardless of the scrapie strain used for infection, showed the same background levels of T-Tau and P-Tau as that measured in the uninfected TauKO and C57Bl mice (Fig 3).

**Fig 4:**
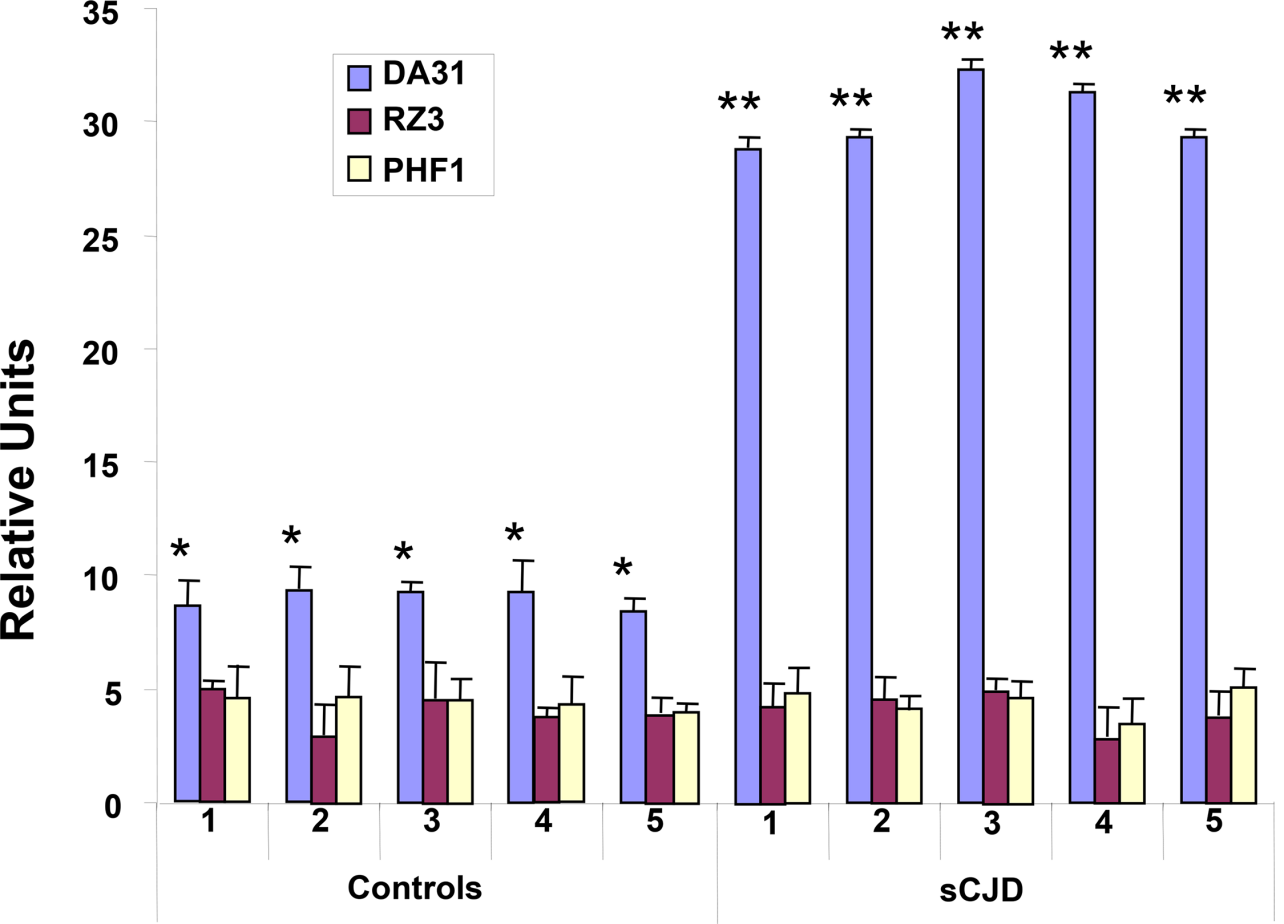
T-Tau (Mab DA31) and P-Tau (Mabs RZ3 and PHF1) levels in normal (controls 1–5) and sCJD (sCJD 1–5) brains measured by EIMAF. Values are expressed as mean ± SD. Significance between T-Tau vs. P-Tau in control samples (*p < 0.05) and between T-Tau levels in control vs. sCJD (**p < 0.01) is determined by the unpaired t-test.

**Fig 5:**
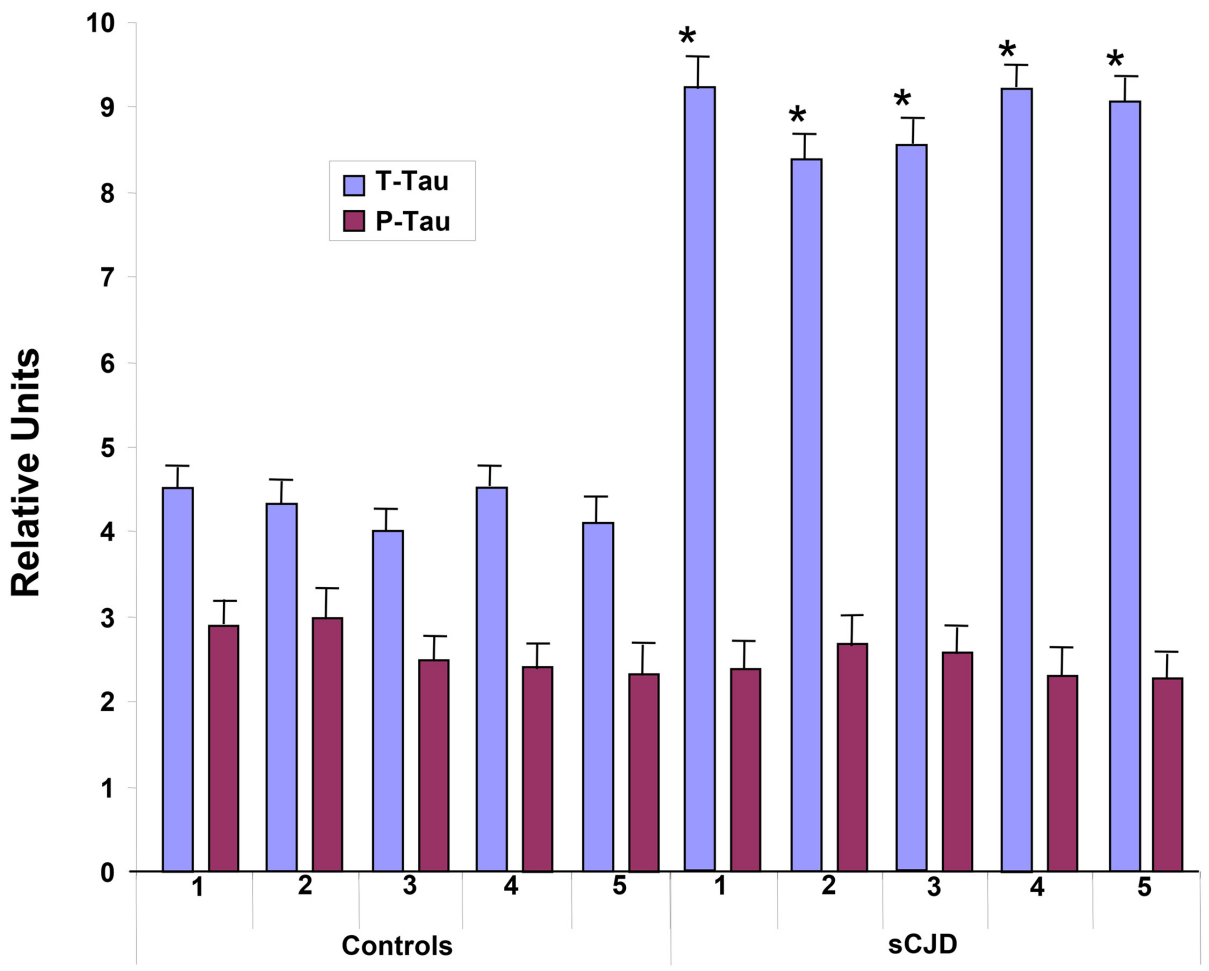
T-Tau (Mab DA31) and P-Tau (Mab RZ3) levels in normal (controls 1–5) and sCJD (sCJD 1–5) plasma measured by a-EIMAF. Values are expressed as mean ± SD. Significance of T-Tau between controls vs. sCJD is determined by the unpaired t-test (*p < 0.05).

Frozen brain and plasma from five human controls and five neuropathologically confirmed human cases of sCJD were assayed for T-Tau and P-Tau as described above. Neuropathologically, all of the sCJD cases had classic morphology and distribution of spongiosis and astrocytosis. Confirmation of sCJD was obtained by immunohistochemical staining and immunoblotting for PrP^Sc^ (data not shown). The levels of T-Tau in the control brains, as measured by EIMAF, were approximately two-fold greater than the P-Tau levels from the same samples but significantly less than the T-Tau from the sCJD brains (Fig 4). Further, the levels of T-Tau in all of the sCJD cases were significantly higher than those found in the control brain samples while, in contrast, the P-Tau values remained unchanged (Fig 4). When compared to the brain, similar general profiles of T-Tau and P-Tau levels were also observed in the blood as determined by a-EIMAF (Fig 5). The levels of P-Tau in sCJD plasma were unchanged and similar to the normal human cases. In contrast, the levels of T-Tau in sCJD were significantly higher than in the normal human plasma samples (Fig 5).

## Discussion

The goal of these experiments was to determine whether the Tau protein in blood, compared to brain, had any use in diagnosis and distinguishing the prion related diseases. To that end, we compared 3 well-characterized mouse adapted scrapie strains and also analyzed sCJD samples from humans. The Tau protein is mainly localized in neurons of the CNS. A major function of Tau is to stabilize and provide an ordered array of microtubules in axons that is necessary for axonal transport. Tau contains 85 potential phosphorylation sites and of those, 71 can be phosphorylated under pathological or physiological conditions. Furthermore, at least 20 different protein kinases have been identified that can utilize Tau as a substrate for phosphorylation. Physiologically, Tau phosphorylation is used to regulate the affinity of Tau for microtubules and also the binding of Tau to signaling molecules. Tau phosphorylation is also regulated during development; there is higher phosphorylation of Tau in the fetal brain which is lower in the adult brain. Coincident with neurodegeneration, Tau is aberrantly phosphorylated and this Tau hyperphosphorylation plays a central role in Tau-mediated toxicity, presumably by reducing its ability to bind to microtubules and consequent destabilization of the microtubules. The increased levels of free unbound Tau can lead to an increase in oligomers, aggregation and fibrillization. In prion diseases, P-Tau deposition has only been found in a few rare cases. In contrast, AD and related disorders are associated with Tau being sequestered into neurofibrillary tangles mainly in neurons. Thus, the loss of Tau's normal function of stabilizing microtubules leads to a pathological disturbance in the normal structure of the cytoskeleton which, in turn, compromises axonal transport, contributing to synaptic dysfunction and neurodegeneration.

In human brain and plasma samples, T-Tau, but not P-Tau, was significantly elevated in clinical sCJD cases compared to normal controls. Our results confirm those reported by others using blood and CSF [26–29]. We further demonstrated that measurements from prion disease blood of not only T-Tau, but both P-Tau and T-Tau and calculation of the P-Tau/T-Tau ratio may be useful for discriminating patients with CJD from individuals with other neurodegenerative disorders based on our observation that P-Tau in sCJD is unchanged, whereas it is elevated in AD [30].

In contrast to the human samples, brain samples from mice infected with the mouse-adapted scrapie strains ME7, 139A and 22L showed elevated P-Tau compared to uninfected mice. In addition, while the human CJD samples exhibited increased T-Tau in the plasma, the infected mice showed essentially unchanged T-Tau and P-Tau in plasma that was indistinguishable from the TauKO mice. Although we expected to detect T-Tau and/or P-Tau in the mouse plasma since the blood-brain barrier is compromised in symptomatic scrapie-infected mice [31], the levels may be too low for detection. In any case, there are a number of possible explanations for the contrasting findings of mouse vs. human Tau. First, the biology of human and mouse prion disorders may not be the same. Second, this result may reflect mass effects; the human brain is a larger proportion of the body than the mouse and consequently more T-Tau may be released into the circulation in humans. Third, the mouse model uses exogenous infection with a brain homogenate, whereas the sCJD cases are believed to arise by spontaneous conversion of endogenous prion protein. This possibility could be examined by analyzing plasma or brain from cases of iCJD. Interestingly, P-Tau/T-Tau ratios might then be useful for diagnosing iCJD where one might expect a scrapie infected murine-like Tau profile rather than a sCJD-like profile. Finally, the antibodies used in the analysis detect phosphorylation at different sites; pThr231 in human and pSer202 in mouse. Cras and colleagues have reported that in CJD there is a difference in phosphorylation at different sites in Tau with pSer202 and pSer404 being more phosphorylated than pThr181, pThr205, and pThr231 [32]. Consequently, resolution of the difference in the mouse and human findings may require a complete analysis of the phosphorylation state of human Tau.

Asuni et al [18] reported an increase in P-Tau staining by immunohisto-chemistry in ME7-infected hippocampus at the late stages of clinical disease. This staining did not localize to the CA1 synapses, the site of synaptic dysfunction in these infected mice, which suggests that Tau is not associated with early disease pathology but instead late-stage disease. Further, transgenic mice overexpressing four-repeat Tau with confirmed impairment of axonal transport demonstrated similar incubation times for clinical disease as compared to control animals [19] again suggesting the lack of influence of Tau on the early progression of disease. However, a Tau-PrP link has been suggested for the human prion diseases, GSS [19, 33, 34] and vCJD [20]. Increased levels of P-Tau in scrapie-infected mice is consistent with increases of P-Tau associated not only with BSE-infected bovinized transgenic mice [35] but also vCJD in humans and experimental mouse models displaying P-Tau positive neurites together with PrP^Sc^ plaques [20]. CSF from a case of sCJD showed significant increases in T-Tau but only a slight increase in P-Tau [36]. Since only 16% of sCJD cases develop plaques a careful study including plaque containing versus plaque negative cases should be performed.

We can conclude that the levels of T-Tau and P-Tau are likely not a contributing factor in the initiation and progression of disease-associated neuropathology of scrapie strain-infected mice. Likewise, it is also likely that Tau cannot be used as a parameter to identify and differentiate scrapie strains. However, as suggested by others the role of Tau phosphorylation in GSS [19] and in human prion diseases initiated by infection (iCJD) may be significant and contrast with its role in human prion diseases where an infectious source is yet to be identified (sCJD).

## Materials and Methods

### Ethics statement

De-identified, archived frozen human brain and blood were obtained from the National Prion Disease Pathology Surveillance Center (NPDPSC) (Case Western Reserve University, Cleveland, OH) (URL: http://www.cjdsurveillance.com/). The SUNY Downstate Medical Center (SDMC) Institutional Review Board, which oversees and protects the use of human subjects and samples in research, granted an exempt status for a full protocol review. All handling, use and disposal of human samples was carried out with the approval of the SDMC Institutional Biosafety Committee. All data obtained in our study were analyzed anonymously.

All animal procedures and sample handling were carried out humanely with approval and oversight from the SDMC Institutional Animal Care and Use Committee (Protocol # 07-251-09) and in accordance with the National Institute of Health Guide for the Care and Use of Laboratory Animals. The handling, use and disposal of animal samples was approved by the SDMC Institutional Biosafety Committee.

### Human samples

Frozen brain and blood from 5 coded cases of sCJD and 5 cases of pathological controls were used. All of the archived sCJD brains were reported by the NPDPSC to have histology and immunohistochemistry consistent with Met/Met polymorphism at codon 129. All cases demonstrated classic distribution of spongiform degeneration with reactive astrocytosis and PrP^Sc^ immunostaining.

### Infection of mice

Weanling (3–4 week old) female C57Bl/6J mice were infected intracerebrally (i.c.) with 50 μl of a 1% brain homogenate (approximately 5 logs of infectivity) prepared from mice who were clinically affected with the mouse-adapted scrapie strains ME7, 139A or 22L (15 mice i.c. injected/scrapie strain). Control age and sex matched mice were sham-infected using a 1% uninfected mouse brain homogenate. Normal brain homogenate was used as the diluent for all inoculums.

### Clinical disease

Mice were monitored twice per week up until 100 days post infection and then daily for clinical symptoms until sacrifice. Clinical symptoms included weight loss, ataxia, hunched posture, ruffled coat and changes in behavior including lack of socialization. The terminal stages were judged to be the time at which animals would die within 48–72 hrs.

### Sample collection

At the terminal stages of disease, mice were placed under deep anesthesia with isoflurane. Blood was collected in heparinized tubes from tail veins or by cardiac exsanguination. Following centrifugation, plasma was obtained, stored at -80° C, and diluted (1:40) prior to use. Brains were removed immediately following euthanasia with isoflurane and stored at -80° C.

### Western blotting

To prepare 10% (wt/vol) brain homogenates for PrP^res^ analysis, mouse brains were homogenized in ice-cold lysis buffer (phosphate-buffered saline [PBS] with 1% Igepal CA-630, 0.5% sodium deoxycholate, 5 mM EDTA, pH 8.0). After centrifugation at 1000 ×g for 10 min, a 100 μl aliquot of the supernatant was treated with proteinase K (PK) (100 μg/ml final concentration, 50° C, 30 min) followed by addition of 1% protease inhibitor cocktail. The sample was then boiled for 3 min with 1x Laemmli sample buffer and a 25 μl aliquot was electrophoresed through a 10% resolving gel and immunoblotted.

For Tau analysis, 10% (wt/vol) mouse brain homogenates were prepared in ice-cold Tris-buffered saline (TBS) (10 mM Tris-HCl, 140 mM NaCl, pH 7.4) containing 10 mM sodium fluoride, 2 mM EGTA, 1mM sodium vanadate and 100x protease inhibitor cocktail. A 0.04 volume of 5 M NaCl and beta-mercaptoethanol to a final concentration of 5% (v/v) were added to the homogenates. The samples were heated to 100° C for 10 min and cooled on ice for 30 min. The samples were microcentrifuged at 10K x g for 15 min at 4° C. Twenty micrograms of each supernatant was treated with 1x Laemmli buffer (100° C, 3 min) and used for Tau analysis by SDS-PAGE on 10% resolving gels.

Proteins were transferred to nitrocellulose membranes overnight at room temperature using 4 volts/cm while submerged in transfer buffer (25mM Tris, pH 8.3, 192 mM glycine and 20% methanol). After transfer, the nitrocellulose was incubated in 5% non-fat dry milk dissolved in PBS-0.1% Tween 20 (PBST) for 30 min. Blots were probed for murine PrP (2 μg/ml Mab 11F12), T-Tau (1:1000 Mab DA9 cell culture media), P-Tau (1:500 Mabs CP13 or PHF1 cell culture media) by incubation in PBS for 2 hrs. Blots were washed 3 times in PBST for 15 min followed by a 1 hr incubation with a 1:2000 dilution of secondary antibody [goat anti-mouse Ig conjugated to either alkaline phosphatase and developed with NBT/BCIP substrate (Life Technologies) or horseradish peroxidase and developed for chemiluminescence using the Supersignal West Dura substrate kit (Thermo Scientific)]. The exposure was recorded using Epi Chemi II Darkroom (UVP Bioimaging) and densitometry performed using Image J software.

### EIMAF and a-EIMAF

The anti-Tau monoclonal antibodies (Mabs) used were previously described [37] and epitopes are indicated below. For enhanced immunoassay using multi-arrayed fiber optics coupled to rolling circle amplification (a-EIMAF), high-binding 96-well microtiter plates (Costar) were coated with capture Mab at 6 μg/ml final concentration [Mab DA31 (aa150-190) for murine and human T-Tau and Mabs CP13 (pSer-202) and PHF1 (pSer-396, pSer-404) for murine P-Tau while human P-Tau was detected with Mabs RZ3 (pThr-231) and PHF1. Following an overnight incubation at 40° C, unoccupied binding sites were blocked for 1 hr with casein. A 100 μl aliquot of diluted (10^−8^) brain or blood (plasma at 1:40 dilution is used to avoid matrix effects) sample was added, incubated and followed by the addition of a biotinylated detection Mab DA9 (aa102-140) (100 μl at 4 μg/ml final Mab concentration). Five 10 min washes with PBST were followed with the addition of 100 μl of streptavidin (5 μg/ml) per well and incubation for 1 hr at 37° C. A biotinylated DNA primer (5'-TTTTTTTGTCCGTGCTAGAAGGAAACAGTTAC-3') (100 μl at 4 μg/ml) was added and the plate incubated for 1 hr at 37° C. Following the addition of a T4-DNA ligase-pretreated IgE DNA template (1 mg/ml), amplification was initiated by adding 100 μl of reaction mixture consisting of: φ29 DNA polymerase reaction buffer, bovine serum albumin, nucleotide triphosphates supplemented with dUTP-Texas Red, and φ29 DNA polymerase. Incubation for several hrs was followed by PBST washes, addition of 1N NaOH, neutralization with 1 M Tris-HCl, pH 7.5, heat treatment (100° C for 15 min) and fluorescence analysis. For direct, non-amplified detection and relative quantitation of Tau, enhanced immunoassay using multi-arrayed fiber optics (EIMAF) was performed as detailed previously and briefly described here [38]. For direct EIMAF, diluted brain or plasma samples were added to the capture Ab followed by the biotinylated detection Mab DA9. Following a 1 hr incubation, streptavidin conjugated to Rhodamine Red X (1:1000) (Invitrogen) was added and incubated for 1 hr. The wells were washed with TBS containing Tween-20, then treated with NaOH and neutralized. A 90 μl sample was drawn up into a 100 μl Microcap (Drummond Scientific) micro-capillary tube, which was then inserted into a specially designed tube sample holder for laser excitation and emission quantitation. Each EIMAF and a-EIMAF sample was tested in triplicate and, depending on available sample volumes, duplicated in independent experiments. Although we produced standard curves to convert voltage to units of P-Tau, we feel that a conversion of voltage to absolute P-Tau levels is inaccurate due to the inefficient levels of recombinant Tau that is phosphorylated artificially *in vitro* and thus we are presenting the data as arbitrary relative units for comparative purposes.
